# PME58 plays a role in pectin distribution during seed coat mucilage extrusion through homogalacturonan modification

**DOI:** 10.1093/jxb/erw025

**Published:** 2016-02-19

**Authors:** Amélie Turbant, Françoise Fournet, Michelle Lequart, Luciane Zabijak, Karine Pageau, Sophie Bouton, Olivier Van Wuytswinkel

**Affiliations:** ^1^Unité Biologie des Plantes et Innovation (BIOPI) EA3900, Université de Picardie Jules Verne, 80039 Amiens, France; ^2^Plateforme d’Ingénierie Cellulaire et Analyses des Protéines, Université de Picardie Jules Verne, 80036 Amiens, France

**Keywords:** Arabidopsis, homogalacturonans, immunolabeling, mucilage, pectins, pectin methylesterase, seed coat.

## Abstract

Pectins are major components of plant primary cell walls. They include homogalacturonans (HGs), which are the most abundant pectin and can be the target of apoplastic enzymes like pectin methylesterases (PMEs) that control their methylesterification level. Several *PMEs* are expressed in the seed coat of *Arabidopsis thaliana*, particularly in mucilage secretory cells (MSCs). On the basis of public transcriptomic data, seven *PME* genes were selected and checked for their seed-specific expression by quantitative reverse transcription PCR. Of these, *PME58* presented the highest level of expression and was specifically expressed in MSCs at the early stages of seed development. *pme58* mutants presented two discrete phenotypes: (i) their adherent mucilage was less stained by ruthenium red when compared to wild-type seeds, but only in the presence of EDTA, a Ca^2+^ chelator; and (ii) the MSC surface area was decreased. These phenotypes are the consequence of an increase in the degree of HG methylesterification connected to a decrease in PME activity. Analysis of the sugar composition of soluble and adherent mucilage showed that, in the presence of EDTA, sugars of adherent mucilage were more readily extracted in *pme58* mutants. Immunolabelling with LM19, an antibody that preferentially recognizes unesterified HGs, also showed that molecular interactions with HGs were modified in the adherent mucilage of *pme58* mutants, suggesting a role of PME58 in mucilage structure and organization. In conclusion, PME58 is the first PME identified to play a direct role in seed mucilage structure.

## Highlight

*PME58* is the first pectin methylesterase gene identified to play a direct role in seed coat mucilage structure. Its activity influences pectin distribution upon mucilage extrusion.

## Introduction

The cell wall plays a key role in numerous aspects of plant biology. From a macroscopic point of view, it is responsible for the plant stature, and its structural modifications throughout evolution have enabled the plant kingdom to conquer the large diversity of terrestrial biomes. However, the presence of this wall prevents cell motion and requires specific mechanisms to achieve cell division and differentiation, which are basic processes controlling important aspects of plant development (Cosgrove, 2005). A widely accepted hypothesis is that plant tissue growth is driven by the internal turgor pressure of cells, particularly during cell elongation, which initiates cell wall loosening and leads to the remodelling of the cell wall followed by the synthesis of new cell wall elements. Despite the fact that the structure and composition of the plant primary cell wall are now well described (Cosgrove, 2005; Caffall and Mohnen, 2009), understanding the molecular mechanisms underlying its modification during cell differentiation, as well as their consequences, is not an easy task. This is because not only is the cell wall a complex dynamic matrix but also the interactions between its various components are difficult to evidence.

The primary cell wall is mainly composed of three families of polysaccharides (cellulose, hemicellulose, and pectins) but also contains proteins, ions, and water. Most of the work concerning the modification of cell wall mechanical properties has focused on the interactions between cellulose microfibrils and hemicelluloses, like xyloglucans, which confer most of its mechanical resistance (Baskin, 2005; Geitmann and Ortega, 2009). Enzymatic activities *in muro* are involved in these modifications (Li *et al.*, 2003). Recent studies showed that modifications of the pectic fraction of the cell wall also participate in the control of plant development (Palin and Geitmann, 2012). Pectins constitute around 35% of the primary cell wall in dicots and non-grass monocots (Mohnen, 2008) and can form a gel-like structure because of their high galacturonic acid content (Jarvis, 1984). They are synthesized in the Golgi apparatus before being secreted into the extracellular matrix by exocytosis (Ridley *et al.*, 2001; Mohnen, 2008; Harholt *et al.*, 2010). 

Pectins are a mix of different polymers, such as type 1 and 2 rhamnogalacturonans (RG-I, RG-II), which are the most complex and highly branched polymers, and homogalacturonans (HGs), which are linear chains of galacturonic acid residues. HGs are the most abundant pectic polymers in the primary cell wall (Caffall and Mohnen, 2009) and are secreted to the cell wall in a highly (70–80%) methylesterified form (O’Neill *et al.*, 1990; Pelloux *et al.*, 2007). In the wall, pectin methylesterase (PME) activity can remove these methyl groups, leading to drastic changes in the physical properties of the polymer (Pelloux *et al.*, 2007; Wolf *et al.*, 2009; Levesque-Tremblay *et al.*, 2015b). In fact, HG de-esterification by PMEs leads to the creation of free carboxylic acid groups that can be involved in Ca^2+^ cross-linking to form a so-called egg-box structure (Micheli, 2001; Pelloux *et al.*, 2007). Thus, at first sight, a low level of HG methylesterification due to PME activity can be associated with an increase in wall rigidity, with consequences on cell growth. This has been shown experimentally on several models, such as pollen tube elongation (Parre and Geitmann, 2005; Bosch and Hepler, 2006), hypocotyl growth (Derbyshire *et al.*, 2007; Pelletier *et al.*, 2010), embryo development (Levesque-Tremblay *et al.*, 2015a), and seed germination (Müller *et al.*, 2013, Scheler *et al.* 2015). However, de-methylesterification of HGs by PMEs, in combination with other enzymatic activities, can also produce an opposite effect, that is, a loosening of the cell wall. As an example, PME activity can regulate organ primordia formation in the shoot apical meristem through a loosening of the cell wall (Peaucelle *et al.*, 2008; Peaucelle *et al.*, 2011). PME activity also contributes to the temporal regulation of the radicle emergence of seeds by altering the mechanical properties of the cell walls (Müller *et al.*, 2013).

As more data become available, interest in better understanding the involvement of PME-coding genes in plant development is increasing (see Sénéchal *et al.*, 2014 for a review). However, this task is facing two main problems. First, PMEs are encoded by a large gene family (66 isoforms in Arabidopsis) (Pelloux *et al.* 2007; Wang *et al.*, 2013) with potential redundancy in their biological functions. Second, the effects of HG methylesterification modifications on the molecular structure of the cell wall show diverse modes of action due to the interactions between HGs and other cell wall polymers, a subject of ongoing study.

To increase knowledge on this topic, the use of simplified cellular models can be of help. For this purpose, mucilage secretory cells (MSCs) present in the epidermal layer of the seed coat of Arabidopsis have several advantages, and, although the composition and structure of mucilage are quite different from those of a typical primary cell wall, it has become a model system to study the plant polysaccharides involved in its construction (Arsovski *et al.*, 2010). For example, the composition and the structure of *Arabidopsis* seed mucilage have been extensively studied because it can be easily extracted (Western, 2012; North *et al.*, 2014). It also contains large amounts of pectins, RG-I being the most abundant. HG is a minor constituent, present in the adherent layer of seed coat mucilage and showing variation in its degree of methylesterification (DM) according to its position in the layer (Macquet *et al.*, 2007).

In a recent work, it was shown that deregulation of PME activity in the mucilage affects mucilage release upon seed hydration (Saez-Aguayo *et al.*, 2013). *PMEI6* codes for an inhibitor of PME and is specifically expressed in seed coat epidermal cells during mucilage polysaccharide synthesis. Regulation of PME activity contributed to the modification of the cell wall of seed coat epidermal cells, or of the HGs contained in the mucilage, to control mucilage release upon hydration. However, the PMEs targeted by PMEI6 activity were not identified and their involvement in this process was indirectly characterized (Saez-Aguayo *et al.*, 2013).

The aim of this work is to better understand the role of PMEs in the structural modification of the cell wall, using mucilage-producing cells as a model. The *PME58* gene is specifically expressed in these cells. Using classic reverse genetics, pectin immunolabelling, and analytical approaches, this work shows that PME58 activity modifies the molecular interactions between HGs and the rhamnogalacturonic fraction of the seed coat mucilage, evidencing a new role of PMEs in the control of the structure of the plant cell wall. The possibility that PME58 is the target of several mucilage-extrusion regulators is discussed.

## Materials and Methods

### Plant material, growth conditions, and mutant genotyping

*Arabidopsis thaliana* (L.) Heynh *pme58–1* and *pme58–2* mutants were isolated from SALK (SIGnAL, USA) T-DNA insertion collections (Salk_014108 and Salk_055262, respectively). Homozygous plants for the T-DNA insertions in the *PME58* gene were identified by PCR. Genotyping PCR reactions were performed using a *PME58*-specific primer and a T-DNA-specific primer left border matching with the left border of T-DNA (see Supplementary Data Table S1 at *JXB* online).

Arabidopsis (ecotype Columbia-0, Col-0) wild type and mutants were grown on 0.5× Murashige Skoog solid medium (Duchefa) containing 1% sucrose and 0.05% MES monohydrate at pH 5.8. Seeds were treated for 2 days at 4°C to synchronize germination, and placed in a PHYTOTRONIC chamber (16-h photoperiod at 120 μmol m^−2^ s^−1^ and 21°C constant temperature) for *in vitro* seedling growth. After 15 days, seedlings were transferred onto soil in a glasshouse (16-h photoperiod at 120 µmol m^−2^ s^−1^, 21°C, and 55% relative humidity) and regularly watered. Siliques were harvested at various developmental stages. Seed lots used in individual experiments were harvested from plants grown simultaneously. Plant material for the expression analysis was harvested in a previously study (Louvet *et al.*, 2006).

### Gene expression analysis

Tissues were ground in liquid nitrogen and, after a preliminary polyphenol and polysaccharide precipitation (Gehrig *et al.*, 2000), RNA was extracted from the supernatant using a hot phenol purification protocol (Verwoerd *et al.*, 1989). Total RNA was treated with the TURBO DNA-free^™^ kit (Applied Biosystem^®^, Life Technologies^™^) according to the manufacturer’s instructions. RT reactions were performed on 3 µg of RNA using the SuperScript^®^ III First-Strand Synthesis System kit (Invitrogen^™^ , Life Technologies^™^) following the manufacturer’s instructions. All cDNA samples were diluted 1/20 before use.

The lack of a *PME58* transcript in *pme58* mutants was checked by semi-quantitative PCR using primers flanking the insertion sites (see Supplementary Data Table S1 at *JXB* online).

For RT-qPCR, the LightCycler^®^ 480 SYBR Green I Master (Roche) was used in 384-well plates in the LightCycler^®^ 480 Real-Time PCR System (Roche). The crossing threshold values for each sample (the number of PCR cycles required for the accumulated fluorescence signal to cross a threshold above the background) were acquired with the LightCycler^®^ 480 software (version 1.5, Roche) using the second derivative maximum method. The primers used are shown in Supplementary Data Table S1. Stably expressed reference genes (*EF1alpha*, *CLATHRIN*, *APT1*), selected using GeNorm software (Vandesompele *et al.*, 2002), were used as internal controls to calculate the relative expression of target genes, according to the method described in Gutierrez *et al.* (2009).

### Analysis of promoter activity

Phusion® Taq polymerase (Finnzymes) was used to amplify 1.4kb upstream of the *PME58* 5′-untranslated region from Arabidopsis Col-0 genomic DNA using specific forward and reverse primers (see Supplementary Data Table S1 at *JXB* online). The amplified fragments were recombined in the pENTR^™^/D-TOPO^®^ entry vector (Invitrogen^™^) using attL1 and attL2 recombination sites. After sequencing, the promoter was recombined upstream of the β-glucuronidase (GUS) and the enhanced green fluorescent protein (eGFP)-coding sequences into the destination vector pKGWFS7.0 (Karimi *et al.*, 2002; Universiteit Gent, http://www.psb.ugent.be/), using LR Clonase (Invitrogen^™^) following the manufacturer’s instructions. *Agrobacterium tumefaciens* C58C1 was transformed by the plasmid and used for subsequent plant transformation. Arabidopsis Col-0 plants were transformed by the floral dip method (Clough and Bent, 1998). T1 transformants were selected on 50 µg mL^−1^ kanamycin and T4 plants were used for the experiments.

Histochemical detection of GUS activity was performed on siliques at different developmental stages. The protocol used was adapted from Sessions *et al.* (1999). Siliques were harvested and incubated in 80% acetone at −20°C for 20min. They were rinsed three times with water and infiltrated under a vacuum for 20min in GUS solution [100mM sodium phosphate buffer, pH 7, 10mM EDTA, 0.1% Triton X100, 0.5mM potassium ferrocyanide, 0.5mM potassium ferricyanide, 1mg mL^−1^ X-gluc (Duchefa Biochemie)]. Next, they were incubated overnight in the dark at 37°C, and then successively rinsed in 70% ethanol solution. They were visualized with a SteREO Discovery.V20 microscope (Carl Zeiss). In these conditions, GUS staining was effective in both the seed coat and the embryo at all silique developmental stages, as verified with several *prom::GUS* transgenic lines available in the laboratory.

The activity of the *PME58* promoter was also studied on seeds at different developmental stages with a confocal microscope (LSM 780, Carl Zeiss). eGFP was excited with a 488-nm argon laser and fluorescence emission was recorded between 493 and 556nm.

### Staining of seeds and siliques

Whole mature seeds were hydrated in liquid or solid media (0.6% agarose) containing 0.01% ruthenium red (RR; Sigma-Aldrich) and visualized with a SteREO Discovery.V20 microscope (Carl Zeiss).

Mucilage of mature dry seeds was extracted by shaking seeds in 50mM EDTA pH 8 for 30min. After two washes in water, mucilage remaining around the seeds was stained with 0.01% RR (Sigma-Aldrich) and visualized with a SteREO Discovery.V20 microscope (Carl Zeiss).

Before toluidine blue staining, siliques were included in a medium-grade LR White resin (Agar Scientific) according to a protocol adapted from Brennan and Harris (2011). Initially, they were fixed for 2h at room temperature under a vacuum in 2% paraformaldehyde (w/v) and 0.1% glutaraldehyde (w/v) in 100mM PIPES buffer pH 7.2. They were dehydrated for 15min in each of a series of ethanol solutions (30, 50, 70, 90, 95, and 100%) and then infiltrated at room temperature for 1h with a medium-grade LR White resin/ethanol solution in a ratio of 1:2 (v/v) then again in a ration of 2:1 (v/v). Then, they were infiltrated with pure resin for 18h before resin polymerization at 55°C for 24h. Samples were cut with a microtome (Leica RM2265, Leica Biosystems) into 500-nm thick sections and transferred to poly-L-lysine-coated slides. These were dried at 55°C for 30min and sections were stained with 0.1% toluidine blue in 0.1M phosphate buffer for 1min. Stained sections were visualized with a light microscope (Nikon-Eclipse-90i, Nikon).

### Scanning electron microscopy

The morphology of mucilage cells of mature dry seeds was investigated by SEM with an environmental high-resolution electron scanning microscope (Quanta 200 FEG, FEI). The microscope was used in low vacuum mode (under a partial vacuum pressure of water) and no preparation of the sample was necessary. The conditions of observation were as follows: acceleration voltage of 20kV, work distance of ~10mm, and work pressure of 0.8–1.4mbar. Measurements of epidermal cell areas were carried out with ImageJ 1.45s software.

### Immunolabelling

Before immunolabelling, the mucilage of mature dry seeds was extracted by shaking seeds in water for 1h. An additional incubation of 1h in 50mM EDTA pH 8 was also performed on some water-extracted soluble mucilage seeds.

For immunolabelling with LM19 and LM20 primary antibodies (PlantProbes, University of Leeds, UK), seeds were rinsed with 1× phosphate-buffered saline (PBS). They were then incubated with 1:10 diluted LM19 or LM20 antibodies (in 1× PBS containing 1% milk powder) at room temperature and 100rpm for 2h. Seeds were washed three times with 1× PBS and incubated with 1:100 diluted secondary antibody [Alexa Fluor® 488 Goat Anti-Rat IgG (H+L), Molecular Probes®, Life Technologies^™^] in the dark at room temperature and 100rpm for 2h. Seeds were washed again three times with 1× PBS, before being labelled with 0.5% Calcofluor (Fluorescent brightener 28, Sigma-Aldrich) in the dark for 10min. After three washes in water, seeds were mounted in Citifluor AF1 (Agar Scientific) and visualized with a confocal microscope (LSM 780, Carl Zeiss). Calcofluor and Alexa Fluor® 488 were excited with a 405-nm diode laser and a 488-nm argon laser, respectively. Fluorescence emission was recorded between 410 and 500nm for Calcofluor and between 500 and 630nm for Alexa Fluor® 488.

For immunolabelling with 2F4 primary antibody (PlantProbes, University of Leeds, UK), seeds were rinsed with a T/Ca/S buffer (0.5mM CaCl_2_, 150mM NaCl, 20mM Tris-HCl, pH 8.2) and incubated with T/Ca/S buffer containing 1% milk powder for 30min. Seeds were then incubated with 1:125-diluted 2F4 antibody (in T/Ca/S containing 1% milk powder) at room temperature and 100rpm for 3h. Seeds were washed three times with T/Ca/S buffer and incubated with 1:50-diluted secondary antibody (Alexa Fluor® 488 Goat Anti-Mouse IgG (H+L), Molecular Probes®, Life Technologies^™^) in the dark at room temperature and 100rpm for 1h. The protocol was the same as described previously for immunolabelling with LM19 and LM20 antibodies.

### Germination assays

Germination assays were performed with mature dry seeds in Petri dishes on a medium containing water and 0.5% agarose. Before being transferred into a PHYTOTRONIC chamber (16-h photoperiod at 120 µmoL m^−2^ s^−1^ and 21°C constant temperature), seeds were incubated for 3 days in the dark at 4°C. The percentage germination was determined after 24h, 48h, and 7 days of incubation in the PHYTOTRONIC chamber. Germination assays under water-limiting conditions were also performed on mature dry seeds according to the protocol of Rautengarten *et al.* (2008).

### Determination of PME activity

PME activity was determined on soluble mucilage, adherent mucilage, and demucilaged seeds according to a protocol adapted from Rautengarten *et al.* (2008). Soluble mucilage was extracted by shaking 30mg of mature dry seeds in 300 µL of extraction buffer (50mM Na_2_HPO_4_, 1M NaCl, 12.5mM citric acid, pH 6.5) at 200rpm for 2h, and collected after a 1-min centrifugation at 2000rpm. After three seed washes, adherent mucilage was extracted in 300 µL of extraction buffer by 20min of shaking (25 movements/min) in a bead mill (Tissue Lyser II, Qiagen®), and collected after a 30-s centrifugation at 2000rpm. The demucilaged seeds were then ground in 300 µL of extraction buffer. The resulting homogenate was shaken at 4°C for 1h, centrifuged at 12 000rpm at 4°C, and the supernatant collected. Protein concentrations of extracted mucilage and seed extracts were then determined using a Pierce BCA Protein Assay kit (Thermo Scientific). For quantification of PME activity, equal quantities of proteins in 20 µL of supernatant were loaded into 5-mm-diameter wells of gel composed of 0.1% of 90% methylesterified citrus fruit pectin (Sigma Aldrich), 1% agarose, 12.5mM citric acid, and 50mM Na_2_HPO_4_, pH 7.0. After an overnight incubation at 37°C, the gels were stained with 0.025% RR for 45min and rinsed several times with water. Stained areas were measured with ImageJ 1.45s software.

### Monosaccharide composition and determination of the degree of methylesterification of HG

Monosaccharide composition was determined on mucilage extracted from 30mg of mature dry seeds in water or 50mM EDTA pH 8. Soluble mucilage was extracted by shaking seeds in 500 µL of water (or EDTA) at 200rpm for 2h, and collected after a 30-s centrifugation at 2000rpm. Seeds were rinsed with 400 µL of water (or EDTA), centrifuged, and the supernatant collected and pooled with the soluble mucilage. After two washes, adherent mucilage was extracted with 300 µL of water (or EDTA) by 20min of shaking in a bead mill (25 movements/min) and collected after a 30-s centrifugation at 2000rpm. Seeds were rinsed with 400 µL of water (or EDTA), centrifuged, and the supernatant collected and pooled with the adherent mucilage. Polysaccharides of mucilage samples (500 µL) were hydrolysed with 500 µL of 8N trifluoroacetic acid at 100°C for 4h, and dried under nitrogen gas. Hydrolysed sugars were dissolved with 1mL of water and acid, and the neutral sugar composition of a 1:10-diluted sample was determined by high-performance anion-exchange chromatography coupled with pulsed electrochemical detection (HPAEC-PAD; Dionex ICS-3000, Dionex Corporation).

The DM of HG was determined on EDTA-extracted adherent mucilage using the GalA concentration measured by HPAEC-PAD and by quantifying the base-released methanol after sample saponification according to Ralet *et al.* (2012).

### Accession numbers

Sequence data from this article can be found in the Arabidopsis Genome Initiative or GenBank/EMBL databases under the following accession numbers: At5g49180 (*PME58*), At1g11590 (*PME19*), At4g03930 (*PME42*), At4g33220 (*PME44*), At5g60390 (*EF1alpha*), At1g27450 (*APT1*), and At5g46630 (*CLATHRIN*).

## Results

### Expression of *PME* genes during seed development

**Fig. 1.**
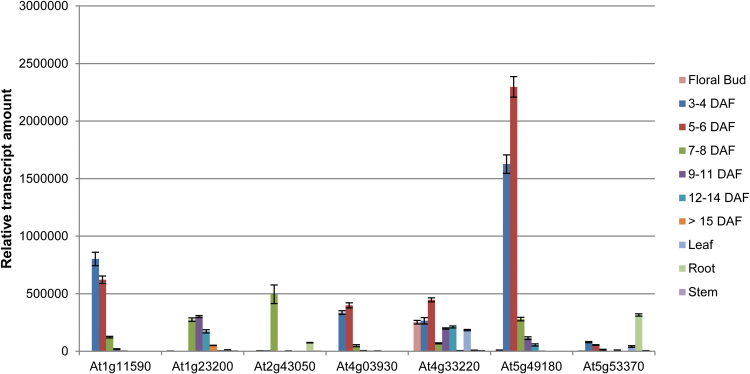
Relative expression of At1g11590, At1g23200, At2g43050, At4g03930, At4g33220, At5g49180, and At5g53370 genes in roots, stem, leaves, flower buds (FB), and siliques at stages 3–4 to >15 days after flowering (DAF). Gene expressions were measured using three stably expressed reference genes (*EF1alpha*, *CLATHRIN*, *APT1*) in two biological samples with similar results. Only results obtained with *EF1alpha* are shown. The presented data correspond to the mean ± SD of two replicates of a biological sample. This figure is available in colour at *JXB* online.

On the basis of public transcriptomic data (http://bar.utoronto.ca/efp/cgi-bin/efpWeb.cgi), seven *PME* genes expressed in seed tegument were selected (At1g11590, At1g23200, At2g43050, At4g03930, At4g33220, At5g49180, and At5g53370). The expression of these genes was analysed by qRT-PCR on total RNA extracted from Arabidopsis siliques harvested at various developmental stages and from vegetative organs. The results confirmed that these seven *PME*s are expressed in siliques, as shown previously in another study (Levesque-Tremblay *et al.*, 2015a). In particular, At1g11590, At1g23200, At4g03930, and At5g49180 are specifically expressed in siliques with very low expression in other organs, such as roots, stem, or leaves (Fig. 1). Of all the *PME*s tested, At5g49180 showed the strongest expression level in siliques, with its transcription peaking at early stages of seed development between 3 and 6 days after flowering (Fig. 1). Interestingly, At5g49180 is also expressed in seed upon imbibition (Scheler *et al.* 2015). Based upon these transcriptomic data, the decision was made to focus on At5g49180, named *PME58*, to characterize its biological function through a classic reverse genetics approach.

### *pme58* mutants present a mucilage phenotype

**Fig. 2.**
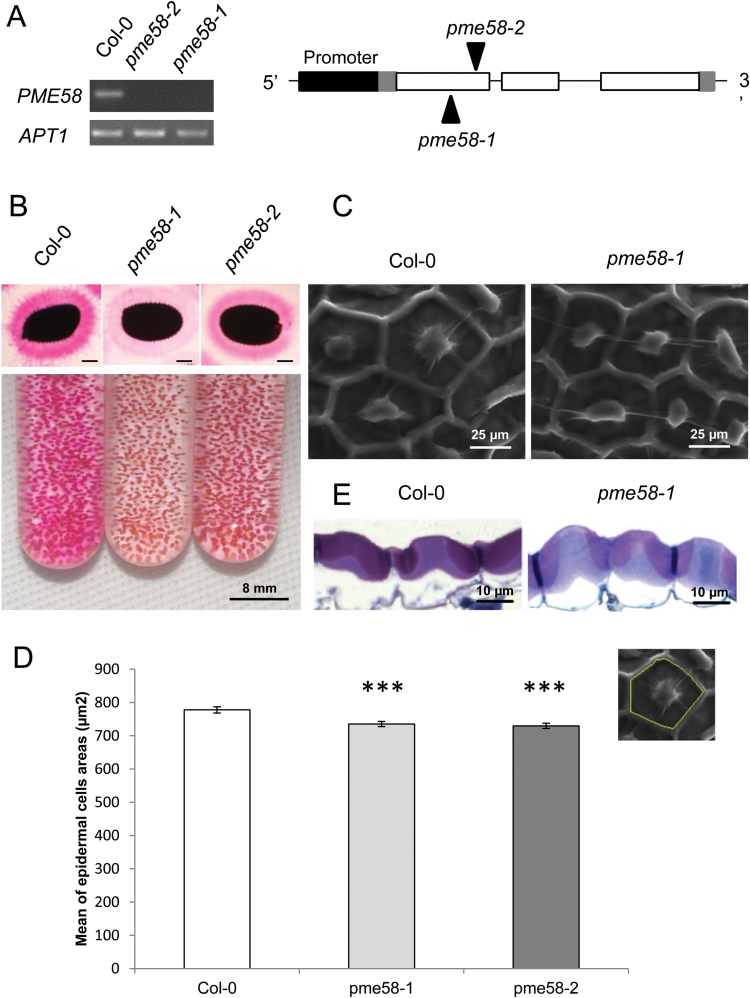
Characterization of T-DNA insertion lines *pme58-1 and pme58-2*. (**A**) Semi-quantitative PCR on cDNA from siliques of wild-type and mutant plants with primers flanking the insertion sites. *APT1* is shown as an internal positive control. (**B**) RR staining of adherent mucilage of wild-type and mutant seeds. Bars = 200 µm. Mucilage remaining around seeds was visualized by RR after extraction in 50mM EDTA pH 8. (**C**) Scanning electron micrographs of the surface of wild-type and *pme58-1* mature dry seeds. (**D**) Epidermal cell surface area (μm^2^), data represent the means ± SE of 600 values obtained on three biological repeats. Significant differences were determined with the parametric T-test (***P < 0.001). (**E**) Sections of mature wild-type and *pme58-1* seeds stained with 0.1% toluidine blue.

Several T-DNA insertion lines affected in the expression of *PME58* were selected and two homozygous mutant alleles were obtained. These two mutants were named *pme58-1* and *pme58-2* and contained a T-DNA insert in the first exon of the *PME58* coding sequence (Fig. 2A). No *PME58* transcript was detected by RT-PCR (Fig. 2A) on either of these lines.

In a first attempt to identify a phenotype, the effect on mucilage extrusion of these two mutant alleles was characterized by RR staining on either liquid or solid medium. The results indicated that *pme58* mutants show a slight phenotype, in that their adherent mucilage layer shows less staining by RR than that of the wild-type Col-0 line, suggesting a possible modification of their mucilage structure. An attempt was then made to characterize the phenotype of *pme58* mutants more precisely.

Arabidopsis adherent mucilage contains a large amount of pectins, mostly RG-I as well as a small amount of HGs. It has been shown that pre-treatment of mucilage with divalent cation chelators, such as EDTA or EGTA, increases the thickness of this adherent layer (Willats *et al.*, 2001). This indicates that the formation of calcium bridges linking the acidic pectic fractions of adherent mucilage plays a role in its molecular structure. To further characterize the adherent mucilage phenotype of *pme58* mutants, their seeds were pre-treated with EDTA prior to RR staining. In these conditions, the adherent mucilage of the *pme58* mutant lines was less stained than that of the wild-type Col-0 (Fig. 2B).

Mucilage is produced in the most external layer of the seed coat. Because PME proteins are located in the plant cell wall, it is possible that the mucilage-producing cell structure is affected in the *pme58* lines. The structure of mutant seed coat epidermal cells was therefore examined in more detail by SEM and toluidine blue staining on mature dry seeds. The epidermal cells of both wild-type and *pme58* mutant seeds had the same morphology, with a characteristic polygonal form and a central columella (Fig. 2C, E). As shown in Fig. 2C, E, the epidermal cells of mutant seeds were smaller than those of wild-type seeds. These observations were confirmed by measuring epidermal cell areas on three biological repeats; they were indeed significantly smaller in *pme58-1* mutants (Fig. 2D) and *pme58-2* mutants (data not shown). So, even if the structure of the epidermal cells of the *pme58* seed coat appears unchanged, a mutation in *PME58* seems to have an effect on epidermal cell size.

Finally, because PME seed activity plays a role in germination (Müller *et al.*, 2013; Scheler *et al.*, 2015), and because *PME58* is strongly expressed in seeds, germination assays were performed under normal and water-limiting conditions after cold stratification. In these conditions, there was no difference in germination between wild-type and *pme58* mutant seeds (Supplementary Fig. S1). We cannot exclude the possibility that seed stratification masks a light effect of *pme58* on germination.

### *PME58* is specifically expressed in mucilage secretory cells

**Fig. 3.**
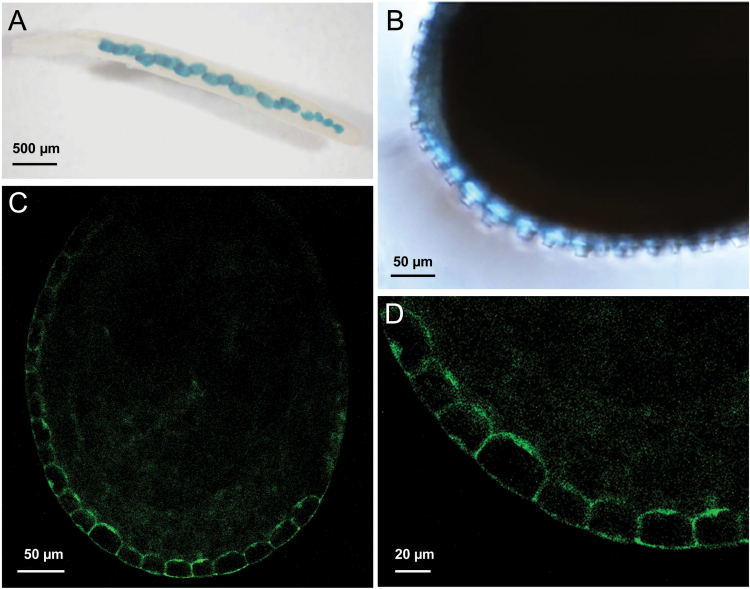
Promoter activity of *PME58*. (**A**, **B**) Histochemical GUS staining of *pPME58:GUS:eGFP* transgenic plants are shown for siliques at stage 2–3 days after flowering (DAF). (**C**, **D**) Confocal laser scanning microscopic analyses of *pPME58:GUS:eGFP* transgenic plants are shown for seed from 7–8 DAF siliques.

Because *pme58* mutants have a mucilage phenotype (Fig. 2B), *PME58* expression was examined to determine whether it is specifically expressed in the seed coat. The activity of the *PME58* promoter was assessed by studying plants transformed with a p*PME58*::*eGFP*:*GUS* construct. As expected, GUS staining showed that the promoter activity of *PME58* is located in the seed coat of young siliques (Fig. 3A) and particularly in the epidermal cells of the seed coat that produce mucilage (Fig. 3B). Moreover, fluorescence of the eGFP protein showed that *PME58* is specifically expressed in mucilage secretory cells (MSCs) of the young seed coat (Fig. 3C, D).

### PME activity and HG methylesterification is modified in *pme58* mucilage

**Fig. 4.**
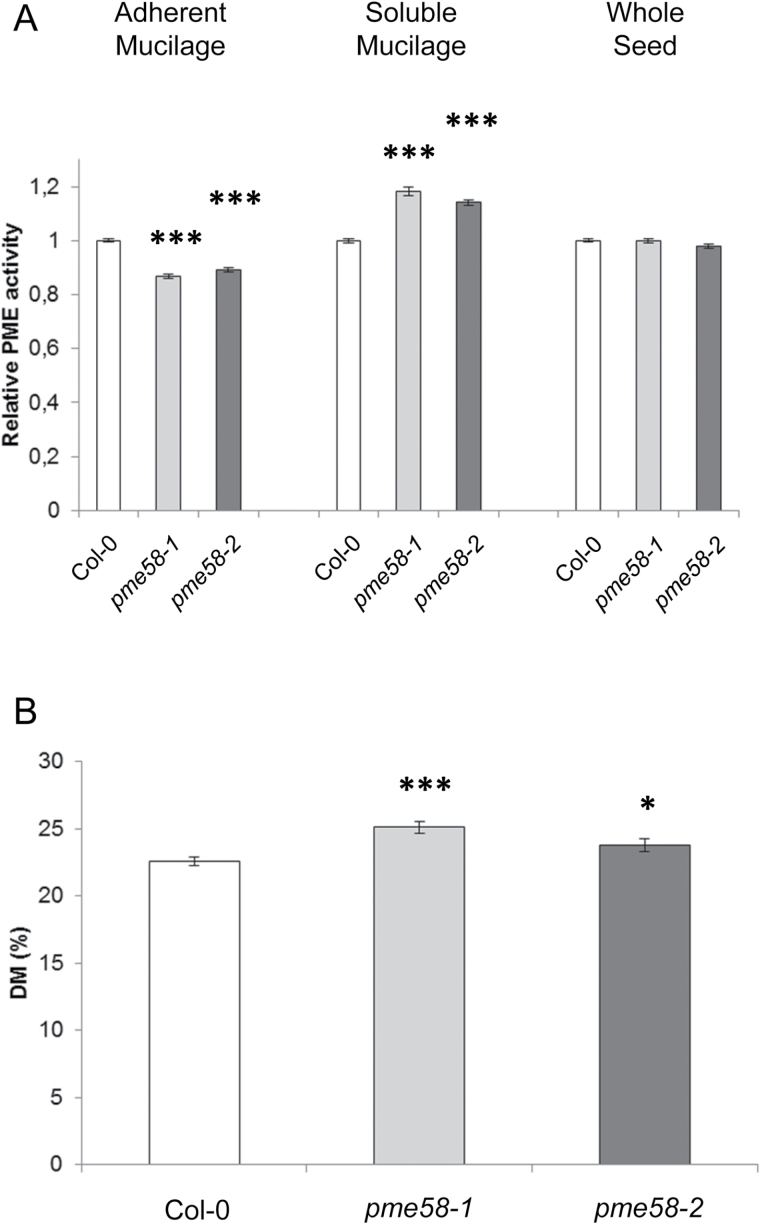
PME activity and DM in wild-type and *pme58* mutants. (**A**) Relative PME activity in adherent mucilage, soluble mucilage, and whole seed determined by gel diffusion. (**B**) Homogalacturonan DM (%) of adherent mucilage. All experiments were performed in triplicate on three biological repeats. Values were obtained from three individual measurements. Data represent the means ± SE of 27 values. Significant differences were determined with the non-parametric Mann-Whitney test (*P < 0.05 and ***P < 0.001).

Because RR is specific to acidic pectins, the decrease in staining observed in *pme58* mutant adherent mucilage after EDTA pre-treatment could indicate a modification of HG methylesterification connected to a decrease in PME activity. Soluble and adherent mucilage were extracted separately on wild-type and *pme58* lines and a PME activity assay was performed on both fractions (Fig. 4A). The measured PME activity was lower in *pme58* adherent mucilage than in the wild type. More surprisingly, PME activity increased in soluble mucilage extracted from the mutant lines. It must be noted that PME activity in the wild type was present in both adherent and soluble mucilage fractions, which are almost equivalent. The difference in PME activity observed in the *pme58* mutant was therefore a direct effect of the mutation. There was no difference between wild-type and mutant lines when PME activity was measured using proteins extracted from demucilaged seeds, confirming that PME58 activity was strictly present in the mucilage-producing cells.

The DM of HGs present in the adherent mucilage was measured on the *pme58* mutant lines and compared to the wild-type lines (Fig. 4B). As expected, the DM was slightly increased in the mutant lines, which had decreased PME activity due to the abolished expression of the *PME58* gene. Although this difference in DM was weak, it was statistically significant.

### Modulation of PME58 activity has an impact on mucilage extractability

**Fig. 5.**
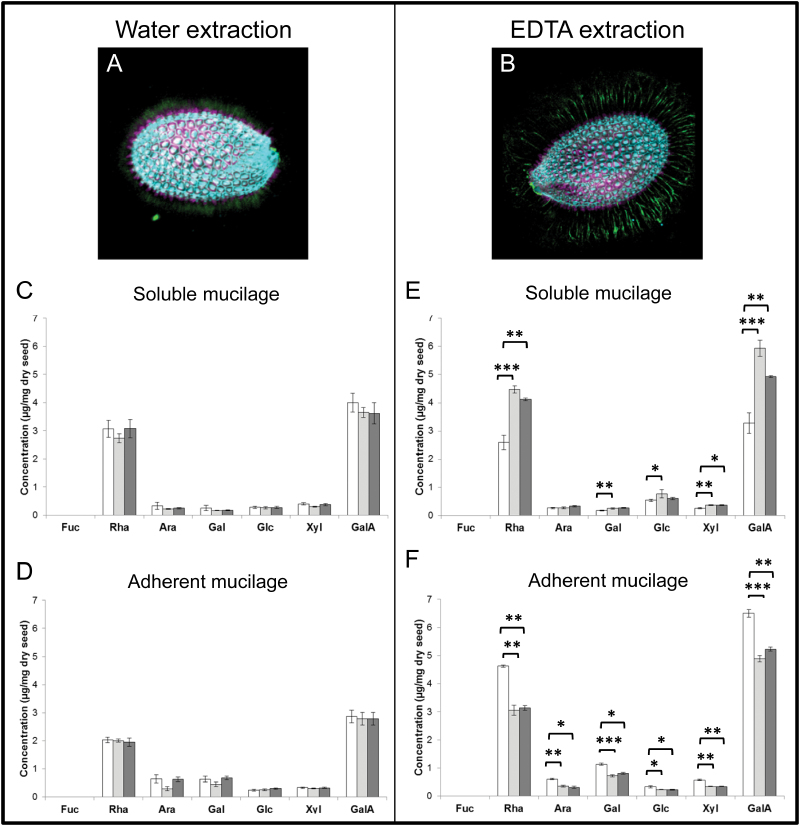
Organization and monosaccharide composition of mucilage extracted in water or EDTA. LM19 immunolabelling of whole seed (green) after soluble mucilage extraction in water (**A**) or 50mM EDTA pH 8 (**B**). Seeds are also labelled with Calcofluor (magenta) and propidium iodide (blue). Calcofluor labels β-glycans, particularly cellulose. Propidium iodide labels cell walls. Monosaccharide composition of soluble (**C**, **E**) and adherent (**D**, **F**) mucilage extracted in water (C, D) or 50mM EDTA pH 8 (E, F) for wild-type (white), *pme58-1* mutant (grey), and *pme58-2* mutant (dark grey) lines. All experiments were performed in triplicate on three biological repeats. Data represent the means ± SE of nine values. Significant differences were determined with the non-parametric Mann-Whitney test (*P < 0.05, **P < 0.01, and ***P < 0.001).

**Fig. 6.**
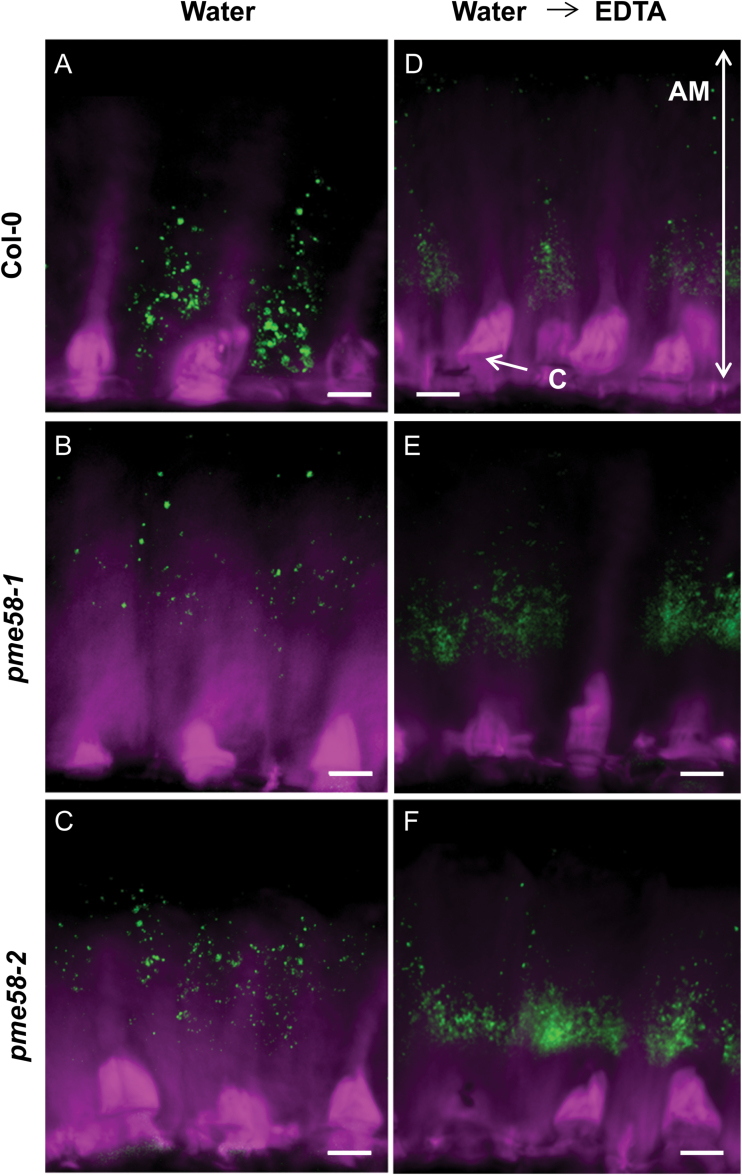
LM19 immunolabelling of adherent mucilage of Arabidopsis seeds. Water-extracted soluble mucilage seeds (**A**, **B**, **C**) were then incubated in 50mM EDTA pH 8 for 1h (**D**, **E**, **F**) before labelling with LM19 (green) and Calcofluor (magenta). Seeds were from either the wild type (A, D), *pme58-1* (B, E), or *pme58-2* (C, F). Bars = 10 µm. AM, adherent mucilage; C, columella.

From the RR staining phenotype observed after mucilage extraction using EDTA (see Fig. 2B), it appears that PME58 activity could influence the molecular structure of the seed mucilage through the modulation of HG DM and its impact on the formation of calcium bridges between HG polymers. It has been shown that HGs demethylesterified by processive PMEs are able to form an egg-box structure through calcium ions binding to the free carboxyl groups generated by this activity, leading to the stiffening of the cell wall pectic structure. Seed mucilage calcium chelation using EDTA has a large impact on the extraction of HGs present in its adherent layer. This can be evidenced by whole seed immunodetection using LM19 antibodies, which preferentially recognize un-esterified HGs (Verhertbruggen *et al.*, 2009).When wild-type seed mucilage was extracted with water, the LM19-specific signal was located at the bottom of the adherent mucilage layer, close to the seed coat surface, as shown by the columella located at the centre of each mucilage-producing cell (Figs 5A and 6A). When seed mucilage was extracted using an EDTA solution, the LM19 signal dispersed to the upper part of the adherent mucilage layer, indicating that the basal location of HGs is highly dependent on the formation of egg-box structures (Figs 5B and 6B). To further study the impact of PME58 activity on the structure of the seed coat mucilage, the free sugar compositions of adherent and soluble mucilage were compared between the wild-type and *pme58* mutant lines after extraction with water or an EDTA solution (Fig. 5C–F). When extracted with water, no difference in mucilage sugar (neutral and acidic) composition could be seen between wild-type and *pme58* mutant lines, in either soluble (Fig. 5C) or adherent (Fig. 5D) mucilage. In both cases, most of the sugars were rhamnose or galacturonic acid, because Arabidopsis mucilage contains mainly RG-I. As expected from previously published results, traces of arabinose, glucose, fructose, and galactose were also detected (Dean *et al.*, 2007; Macquet *et al.*, 2007; Saez-Aguayo *et al.*, 2013; Voiniciuc *et al.*, 2013; North *et al.*, 2014; Yu *et al.*, 2014). However, when extracted with an EDTA solution, mucilage sugar composition was clearly different between wild-type and *pme58* mutant lines, for both soluble and adherent fractions (Fig. 5E, F). In EDTA-extracted soluble mucilage, higher levels of all the detected sugars were present in the *pme58* mutant lines than in the wild type (Fig. 5E). Interestingly, the opposite was seen for the EDTA-extracted adherent mucilage (Fig. 5F). In this case, all the detected sugars were present in lower amounts in the mutant lines than in the wild type. Moreover, the amount of sugar extracted using EDTA was higher than the amount extracted with water, probably indicating that EDTA treatment results in the extraction of pectic sugars not only from mucilage but also from the cell wall of the mucilage-producing cells. Taken together, these results indicate that, upon EDTA extraction, sugars are more easily transferred from the adherent layer to the soluble mucilage fraction in the *pme58* mutant lines, evidencing that PME58 activity participates in the structuration of the mucilage adherent layer.

### PME58 activity modulates molecular interactions with HGs in the mucilage adherent layer

Modulation of the extractability of mucilage in the *pme58* mutants seems to indicate that modification of the DM of HGs present in its adherent layer can influence the molecular interactions between the mucilage components during its extrusion. Immunodetection assays were therefore performed on the mucilage adherent layer to study the effect of PME58 activity on HG location upon mucilage extrusion in different experimental conditions. Following mucilage extrusion in water, the soluble layer was eliminated and immunolabelling was performed on the adherent layer using the LM19 antibody. As expected, the LM19 signal in the wild type was located at the basal of the mucilage adherent layer, close to the seed coat surface (Fig. 6A). However, when the experiment was performed in the same conditions on both the *pme58* mutant lines (Fig. 6B, C), the LM19 signals were found in the upper portion of the adherent layer, clearly above the tip of the columella surface. The experiment was performed several times on different seed samples, giving the same result. When the labelling experiment was performed after mucilage water extraction and elimination of the soluble layer immediately followed by EDTA treatment on only the adherent layer, the LM19 signals were again found to have shifted to an upper position in the mucilage adherent layer of the wild type (compare Fig. 6A, D). In contrast, this additional EDTA treatment had no effect on the LM19 labelling in either *pme58* mutant line (Fig. 6E, F). The same set of experiments was performed using antibodies recognizing other HG epitopes: in the same conditions described for Fig. 6, immunolabelling was performed with the LM20 antibody (which binds to moderately and highly methylesterified HGs) and 2F4 antibody (which binds to HG egg-box structures), and no differences were observed between wild type and mutants in either mucilage extraction condition (see Supplementary Figs S2 and S3 at *JXB* online). These results indicate that PME58 activity participates in the regulation of interactions between HGs and other polymers (probably RG-I) present in the mucilage adherent layer during extrusion.

## Discussion

Despite the amount of data obtained on its composition and synthesis, the plant cell wall is still poorly understood in terms of its dynamics during plant development, especially during basic processes like cell elongation and organ emergence. Covalent and non-covalent interactions between the cell wall components are believed to play a crucial role in establishing its structure and strength (Vincken *et al.*, 2003; Vorwek *et al.*, 2004; Cafall and Mohnen, 2009; Burton *et al.*, 2010). Consequently, understanding the modulation of these interactions could be of major importance in establishing the influence of the cell wall on plant development or, for example, in response to environmental stress conditions. The most recent models of plant cell wall structure describe a cellulose–hemicellulose network surrounded by a pectic gel fraction (Vorwek *et al.*, 2004; Cafall and Mohnen, 2009). The importance of the interactions between pectins and the other cell wall components has been evidenced by several studies (Zykwinska *et al.*, 2005; Popper and Fry, 2008; Wang *et al.*, 2012). However, the nature of these interactions remains unclear. In this context, the use of adapted cellular models to understand these processes is essential, even if these models will never fully reflect the expected complexity of these interactions. In recent publications, the use of MSCs as such a model has been extremely effective in revealing the nature of molecular interactions between cell wall components (Griffiths *et al.*, 2015; Voiniciuc *et al.*, 2015). This model has also been used to show that modulation of HG methylesterification occurs during MSC differentiation and plays a role in controlling mucilage release (Rautengarten *et al.*, 2008; Saez-Aguayo *et al.*, 2013; Voiniciuc *et al.*, 2013). Identifiying the PME genes involved in this model and studying their impact on cell wall or mucilage structure could further our understanding of the complex interactions between pectins and other cell wall components.

### PME58 is specifically expressed in mucilage secretory cells

Despite the apparently large number of *PME* genes expressed in the seed coat (Levesque-Tremblay *et al.*, 2015a), it is still unclear which of these PMEs modify mucilage structure. However, it is known that PME activity is an important regulator of Arabidopsis mucilage secretion. For example, it is known that *PMEI6*, coding for a PME inhibitor protein, is involved in Arabidopsis mucilage release because *pmei6* mutants have impaired mucilage extrusion upon hydration (Saez-Aguayo *et al.*, 2013). Moreover, the subtilisin-like protease SBT1.7 (Rautengarten *et al.*, 2008) is also involved in mucilage release, given that this is defective in the corresponding mutants. Both PMEI6 and SBT1.7 act on this phenotype by modulating PME activity, though with different modes of action. PMEI proteins inhibit PME activity through direct protein–protein interactions while subtilisin protease acts on PME activity at a post-translational level (Wolf *et al.*, 2009; Sénéchal *et al.*, 2014). Interestingly, it seems that PMEI6 and SBT1.7 control different sets of PME proteins in the MSC, given that the double mutant presents additive phenotypes (Saez-Aguayo *et al.*, 2013). The impact of modulating the level of mucilage pectin methylesterification was demonstrated by the identification of FLY1, a transmembrane ring E3 ubiquitin ligase (Voiniciuc *et al.*, 2013). The *fly1* mutant has a lower DM and defective mucilage extrusion as well as increased mucilage adherence. However, the proteins targeted by PMEI6, SBT1.7, and FLY1 have not yet been identified.

To identify the *PME* genes potentially involved in cell wall modification during MSC differentiation and to select for potential targets of PMEI6, SBT1.7, and FLY1, *PME* genes potentially expressed in the seed coat were selected from public transcriptomic data and their expression kinetics were characterized by qRT-PCR during silique development (Fig. 1). Most of the selected *PME* genes showed silique-specific expression, confirming data obtained in a previous study performed using semi-quantitative RT-PCR (Levesque-Tremblay *et al.*, 2015a). However, the data presented in this study also show that the expression level of each *PME* gene is much more diverse than previously determined, and have precise developmental kinetics. Among the selected *PME* genes, *PME58* showed by far the highest level of expression. Moreover, promoter–reporter constructs showed that *PME58* was specifically expressed in the mucilage-producing cells of the seed coat. This result is not in agreement with the data obtained by Levesque-Tremblay *et al.* (2015a), which showed *PME58* expression in both the seed coat and the embryo using a different experimental approach. Consequently, *PME58* was considered the best candidate among the *PME* genes to demonstrate the direct role of PMEs in modifying the MSC cell wall to impact mucilage extrusion.

### *pme58* mutants show two discrete structural phenotypes

Although phenotypes are hard to demonstrate in *pme* mutants, probably because of gene redundancy (Levesque-Tremblay *et al.*, 2015b), a classic reverse genetics approach was used to characterize the biological function of PME58. Two *PME58* knockout homozygous mutant lines were obtained (*pme58-1* and *pme58-2*) and neither showed a mucilage extrusion phenotype. This is not surprising because such a PME-related phenotype has previously been observed when PME activity increases in mucilage-producing cells, as shown in the *pmei6* mutant (Saez-Aguayo *et al.*, 2013). In addition, because PME activity plays a role in the promotion of seed germination (Scheler *et al.*, 2015), whether PME58 could be involved in this process was investigated. *pme58* germination levels were compared to those of wild type but no differences were observed (see Supplementary Fig. S1 at *JXB* online).

*pme58* mutants show two discrete phenotypes. First, the mucilage-producing cell surface area was slightly smaller in *pme58* mutants, as demonstrated by careful statistical analysis of two separate batches of seeds. This could indicate that PME58 is involved in the differentiation process of these cells, probably by modulating the rigidity of the cell wall. Interestingly, a reduction in cell size was also observed in the mutant for the *HMS* gene coding for a PME expressed in the Arabidopsis embryo (Levesque-Tremblay *et al.*, 2015a). It must be noted that, in the currently used model (Pelloux *et al.*, 2007), the decrease in PME activity measured in the *pme58* mutant should lead to a loss of pectin interactions due to the lower formation of egg-box structures, which would result in the loosening of the cell wall. This is not compatible with the phenotype observed, indicating that the impact of the degree of HG methylesterification on cell wall rigidity is probably more complex than expected.

Second, RR staining of adherent mucilage was clearly decreased in the mutants compared to the wild type after EDTA treatment (Fig. 2B). This difference was not as obvious when mucilage extrusion was performed with either water or a CaCl_2_ solution. Although its molecular interactions are not well known, RR is commonly described as a stain for demethylesterified pectins that contain a large number of carboxyl acidic groups. This is particularly the case for PME demethylesterified HGs. Weaker RR staining in the *pme58* mutants could indicate that the pectins contained in the adherent mucilage of the mutants, in which a decrease in PME activity is expected, had a higher DM. In support of this, PME activity was indeed lower in the adherent mucilage of both *pme58* mutants (Fig. 4A) and the DM of the HGs was higher (Fig. 4B). These straightforward conclusions must be tempered by two additional results. First, the decreased RR staining of adherent mucilage in *pme58* mutants was only visible after EDTA extraction. This may reflect the fact that, in these experimental conditions, RR staining was not specific to HG pectins in the presence of divalent ions, like Ca^2+^, in the mucilage. This ‘background’ aspecific staining was abolished upon EDTA treatment, which allowed the specific detection of demethylesterified HG in these conditions. Another explanation for these results could be that the amount of acidic pectins was lower in the adherent mucilage of *pme58* mutants upon EDTA extraction. Second, while decreased PME activity was observed in the adherent mucilage of mutants, increased activity was detected in their soluble mucilage. One interpretation of this surprising result is that the decrease in PME activity observed in the adherent mucilage of mutants was caused not only by the mutation but also by the increased migration of other PME enzymes towards the most external layer of mucilage. This effect was only observed in the *pme58* mutants and could indicate that modification of the mucilage structure due to the mutation enabled the mucilage proteins to diffuse at a higher rate in this system. Although no direct proof for this hypothesis was obtained in this work, the overall results clearly show that the absence of PME58 expression in mucilage-producing cells leads to some modification of the mucilage structure through a change in the molecular interactions between HG and other components of the adherent mucilage. Consequently, PME58 is potentially the first seed coat PME enzyme shown to play a direct role in seed mucilage structure.

### PME58 activity modulates the molecular interactions between HG and other mucilage components

A decreased amount of RR staining in adherent mucilage was the main phenotype observed in both *pme58* mutants. To examine the molecular process involved in this phenotype, additional experiments were performed using HG immunodetection and mucilage monosaccharide composition analysis.

Immunodetection experiments using antibodies recognizing different types of HG were performed on whole seed adherent mucilage. Labelling of low-methylesterified HG using LM19 antibody showed a shift in HGs from a basal position in the wild type to a higher position in the adherent mucilage in both *pme58* mutants (Fig. 6). The experiment was repeated several times and always showed these same shifts, despite differences in signal strength between the assays. In the experimental conditions used, shifts in label location were never observed when using other anti-HG antibodies, such as LM20 and 2F4. Interestingly, epitopes recognized by LM20 and 2F4 are mainly located in the cell wall of the MSC and little is in the mucilage structure itself. In contrast, LM19 labels were mainly detected in the adherent mucilage structure after extraction. Finally, mucilage extraction with water followed by EDTA treatment led to the same shift in LM19 signal but only in the wild type, whereas EDTA treatment had no effect on the signals observed in *pme58* mutants. These results indicate that PME58 activity is involved in regulating the interaction of HG in the adherent mucilage with other polysaccharides, and that this interaction requires cross-linking involving divalent ions, probably Ca^2+^. Alternatively, these results could indicate that fewer egg-box structures in the *pme58* mutants result in easier water extraction of HG from the basal cell wall during mucilage extrusion, whereas the wild type requires EDTA to obtain equivalent HG extractability. LM19 labelling in the wild type during whole seed immunodetection clearly showed that EDTA treatment had a strong impact on HG extractability, further confirming that ionic cross-linking is an important factor in HG extraction during mucilage extrusion (Fig. 5A, B).

To verify that PME58 activity is involved in the modulation of mucilage component extractability, the monosaccharide composition of extracted adherent and soluble mucilage was measured. Upon mucilage water extraction, there was no difference in the monosaccharide composition of either adherent or soluble mucilage between wild type and *pme58* mutants (Fig. 5C, D). However, there were obvious differences in mucilage monosaccharide composition between wild type and *pme58* mutants when mucilage was directly extracted using an EDTA solution. Interestingly, the differences between adherent and soluble mucilage were in complete contrast: the amount of all measured monosaccharides was lower in the mutants than in the wild type when considering adherent mucilage composition, but *vice versa* in soluble mucilage (Fig. 5E, F). The clear interpretation of these results is that using EDTA improves mucilage polysaccharide extraction in the *pme58* mutants, indicating again that the link between mucilage polysaccharides is weaker when PME58 activity is altered. This modification of polysaccharide linkages, especially those involving RG-I, could explain the weaker RR staining of the adherent mucilage of *pme58* mutants when compared to wild type after EDTA treatment (Fig. 2B). As mentioned previously, RR is specific to acidic pectins, including HG but also RG-I, the most abundant polysaccharide in Arabidopsis mucilage. Therefore, the strong RR staining of wild-type adherent mucilage is probably mainly due to the labelling of RG-I , whereas the weaker staining in *pme58* mutants could be explained by RG-I migration from adherent to soluble mucilage (Fig. 5E, 5F). Such differences in the distribution of polysaccharides between adherent and soluble mucilage have previously been reported in several mutants with pectin modifications, described below.

The first group of mutants, such as *cesa5*, *fei2*, and *sos5*, shows a decreased amount of adherent mucilage (Harpaz-Saad *et al.*, 2011; Mendu *et al.*, 2011; Sullivan *et al.*, 2011; Harpaz-Saad *et al.*, 2012). In these mutants, the synthesis of cellulose is affected, as evident from the structure of the adherent mucilage, which has a low pectin content. The second group of mutants has the opposite phenotype, with an increased amount of pectins in their adherent mucilage and a decreased amount in their soluble fraction. For example, in the *mum2* mutant, the RG-I content in adherent mucilage is increased because of the decreased trimming of RG-I galactan side-chains by MUM2 β-galactosidase activity (Dean *et al.*, 2007; Macquet *et al.*, 2007). The mutant phenotypes in some of this second group are directly related to modified PME activity. The *sbt1.7* mutant, for example, has increased PME activity, while the amount of soluble mucilage extracted in the presence of EDTA is decreased (Rautengarten *et al.*, 2008). *pmei6* and *fly1* mutants also have increased PME activity and a displacement of pectins from soluble to adherent mucilage (Saez-Aguayo *et al.*, 2013; Voiniciuc *et al.*, 2013). In the case of *pme58* mutants, the measured decrease in PME activity is linked to an increased pectin content in the soluble mucilage but only upon extraction with EDTA. This result indicates that the modulation of HG methylesterification by specific PME activity plays a direct role in regulating the interactions between mucilage pectic polysaccharides and probably cellulose microfibrils. The nature of these interactions is unknown but involves di-cationic agents such as Ca^2+^. The situation is unclear because the increase in HG methylesterification measured in the *pme58* mutant should lead to decreased Ca^2+^ binding to HGs, thus to a decreased interaction between mucilage polysaccharides. However, Ca^2+^ ions still play a role in these interactions in the *pme58* mutant, even in the presence of methylesterified HG, as demonstrated by the fact that a difference in RG-I distribution was only observed upon treatment with EDTA. Because the *pme58* phenotype is basically the opposite of the phenotypes observed in *pmei6*, *sbt1.7*, and *fly1* mutants (with regards mucilage pectin distribution), these results could indicate that PME58 is inhibited by PMEI6 and is the target of the subtilisin protease encoded by SBT1.7 and FLY1 Ring E3 ubiquitin ligase. This should be addressed in further investigations, although these will be limited by the complexity of the system involved in MSC differentiation.

## Supplementary Data

Supplementary materials are available at *JXB* online.

Table S1. List of primer pairs used during the study.

Figure S1. Germination level under normal and water-limiting conditions.

Figure S2. LM20 immunolabelling of adherent mucilage of Arabidopsis seeds.

Figure S3. 2F4 immunolabelling of adherent mucilage of Arabidopsis seeds.

## Funding

This work was supported by the French Ministry of Research (MESR).

## Supplementary Material

Supplementary Data
