# Supplementary material for: PME58 plays a role in pectin distribution during seed coat mucilage extrusion through homogalacturonan modification
Source: J Exp Bot. 2016 Feb 19;67(8):2177–90. doi: 10.1093/jxb/erw025 (PMC4809284; doi:10.1093/jxb/erw025)
Supplement: Supplementary Data [file supp_67_8_2177__index.html]

PME58 plays a role in pectin distribution during seed coat mucilage extrusion through homogalacturonan modification — PME58 plays a role in pectin distribution during seed coat mucilage extrusion through homogalacturonan modification — Supplementary Data 

# PME58 plays a role in pectin distribution during seed coat mucilage extrusion through homogalacturonan modification

## Supplementary Data

Data files

- supplementary\_table\_S1\_figures\_S1\_S3.pdf - Supplementary Data
