# Supplementary material for: PME58 plays a role in pectin distribution during seed coat mucilage extrusion through homogalacturonan modification
Source: J Exp Bot. 2016 Feb 19;67(8):2177–90. doi: 10.1093/jxb/erw025 (PMC4809284; doi:10.1093/jxb/erw025)
Supplement: Supplementary Data [file supp_erw025_supplementary_table_S1_figures_S1_S3.pdf]

Table S1

Experiment	Forward (5'→3')	Reverse (5'→3')
**qRT-PCR**		
At1g11590	CGCTCAGTCACGCGCATTTAAG	TCCGAGCTTGCAGTAATGTTGC
At1g23200	ACATCTACGCTCGTAAGCCGATG	TTGCGTGATTGAGCGGTGATCG
At2g43050	TCCGCCGTCGTATTTCAATCCTG	TTGCGCCGTCACGAAGTTTCTC
At4g03930	CCTTGGGAAGGAGAAACTGGTCTG	TCACCCTTCTGCTAGTCACAGCTC
At4g33220	CACTTTCCGATCAGCTACATTCGC	ATCTCAGTGCTACCGCCTGATG
At5g49180 (PME58)	ACATACCTTACTGCCACTGTTGCG	AAATCCGCCGAGACTCTTAGGG
At5g53370	TTTCACACCGCCACTTTCGC	TTCTCAAACGTCATGTCCCTCAC
EF1α	TGGTGACGCTGGTATGGTTA	TCCTTCTTGTCCACGCTCTT
APT1	GAGACATTTTGCGTGGATT	CGGGGATTTTAAGTGGAACA
Clathrin	GTTTGGGAGAAGAGCGGTTA	CTGATGTCACTGAACCTGAACTG
***PME58* Promoter Cloning**	CACCCCAATCGAAGCCAATCATCT	GAGCCCCTTGTGACTCTGTT
***pme58* mutants Genotyping**		
PME58	GCTTAGTGGCTCCATTGCTT	GCAACCACCACGTTAGCTTT
SALK T-DNA	CGATTTGGAACCACCATCAAACAGGA	TAAAACGGCTTGTCCCGCGTCATC
**Semi q-PCR (*pme58* mutants)**	GCCATATTACCGGAGAACCA	CATCCCGCTGGATCAATAAC

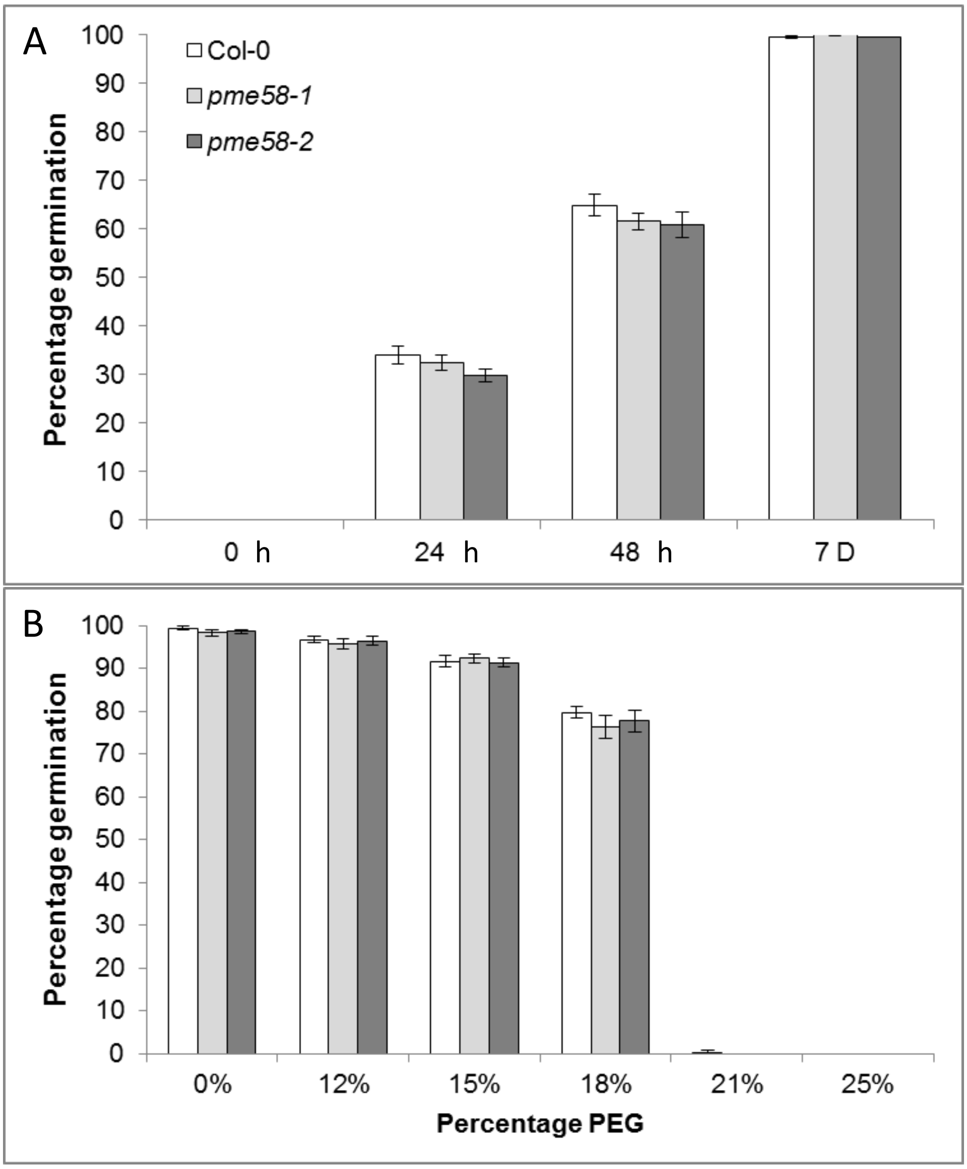

Figure S1. Germination level under normal and water-limiting conditions. Comparison of germination in Col-0, *pme58-1* and *pme58-2* seeds after 24 h, 48 h and 7 days of incubation under normal water conditions (A) and after 5 days on increasing concentrations of polyethylene glycol (PEG, B).

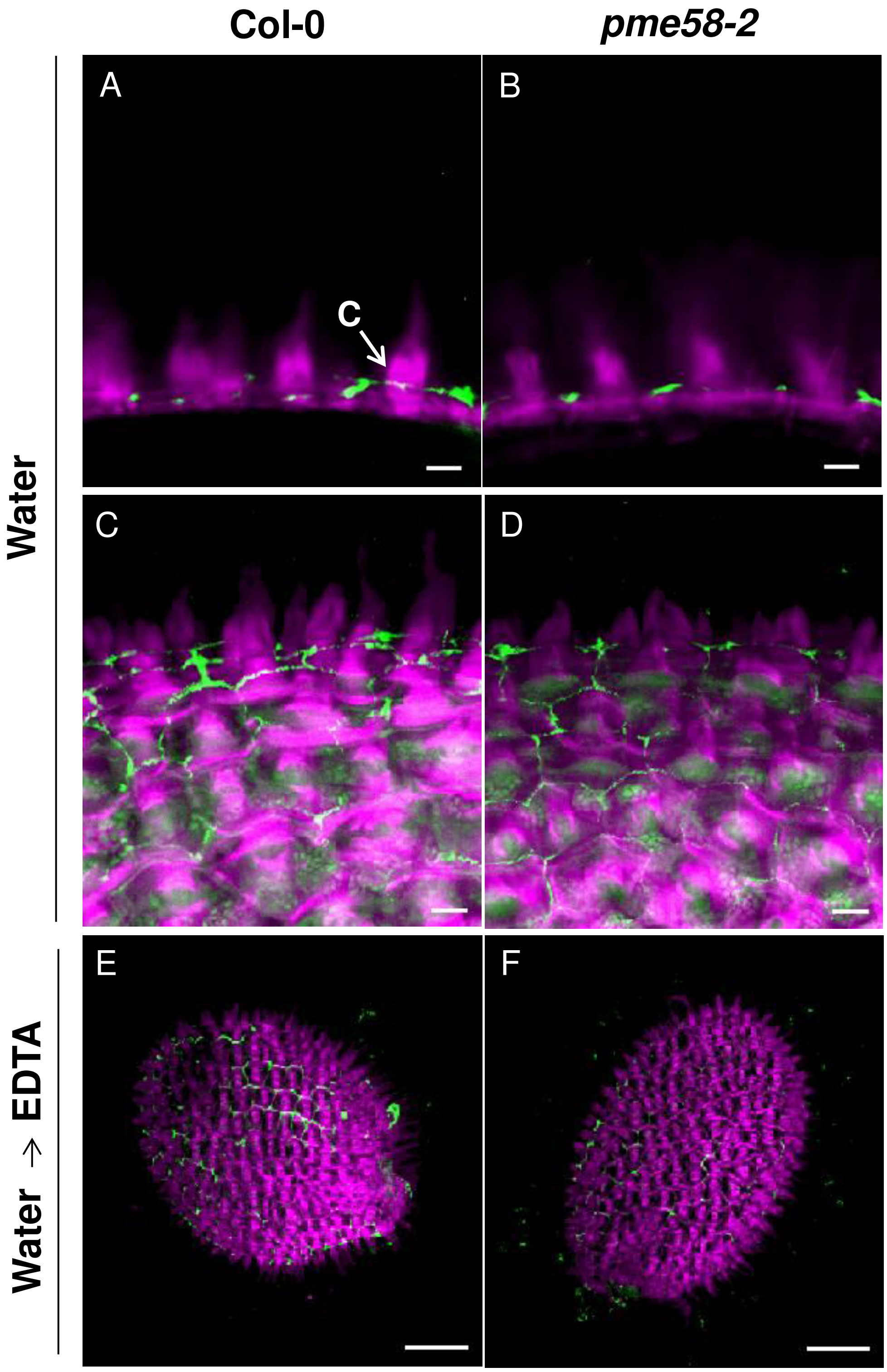

Figure S2. LM20 Immunolabeling of adherent mucilage of Arabidopsis seeds. Water-extracted soluble mucilage seeds (A, B, C, D) were then incubated in 50 mM EDTA pH 8 for one hour (E, F) before labeling with LM20 (green) and calcofluor (magenta). Seeds were from either the wild type (A, C, E) or *pme58-2* (B, D, F). Optical sections through the middle of seeds (A, B) and maximum intensity signals from multiple optical stacks (C, D, E, F). Bars A, B, C, D = 10 µm. Bars E, F = 100 µm. C, columella.

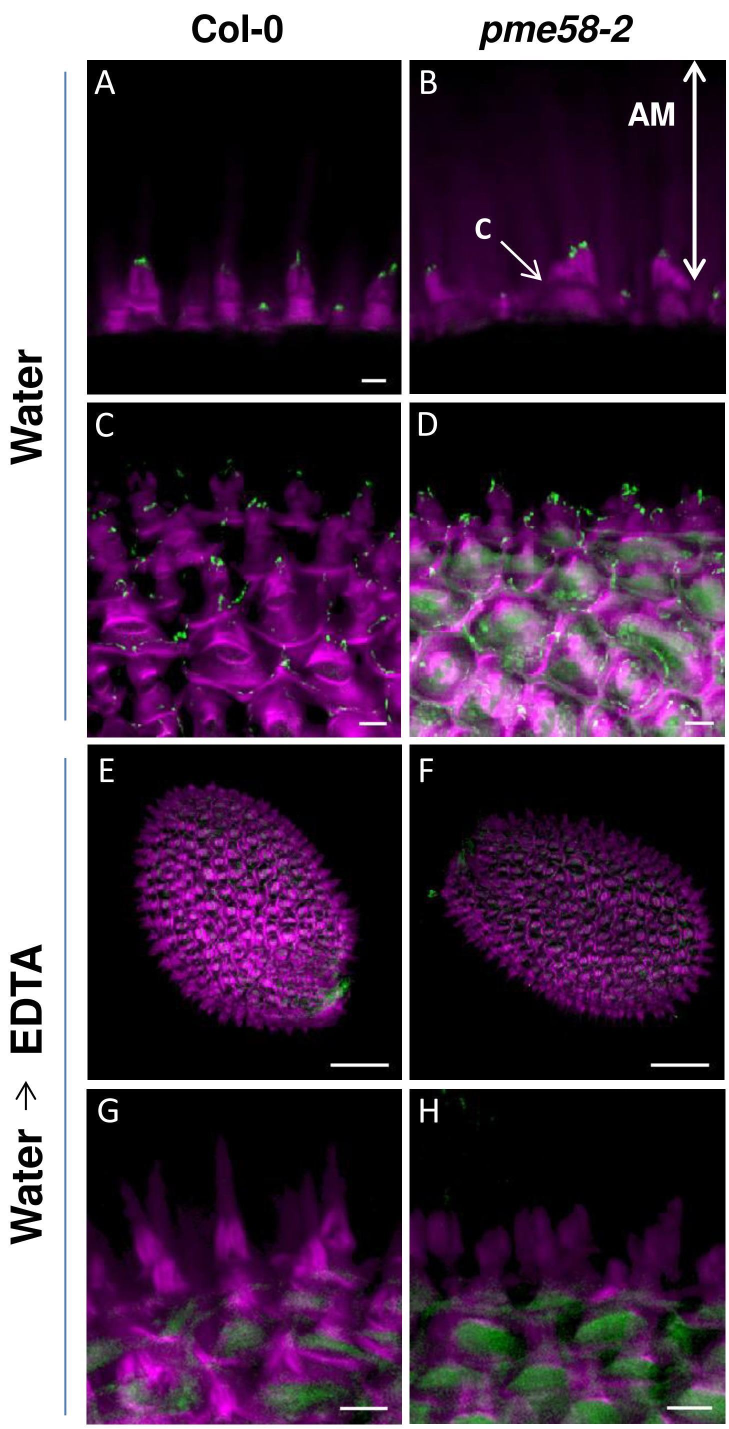

Figure S3. 2F4 Immunolabeling of adherent mucilage of *Arabidopsis* seeds. Water-extracted soluble mucilage seeds (A, B, C, D) were then incubated in 50 mM EDTA pH8 for one hour (E, F, G, H) before labeling with 2F4 (green) and calcofluor (magenta). Seeds were from either the wild type (A, C, E, G) or *pme58-2* (B, D, F, H). Optical sections through the middle of seeds (A, B) and maximum intensity signals from multiple optical stacks (C, D, E, F, G, H). Bars A, B, C, D, G, H = 10 µm. Bars E, F = 100 µm. C, columella and AM, adherent mucilage.
